# Comparison of Femtosecond Laser-Assisted Cataract Surgery and Conventional Phacoemulsification in Shallow Anterior Chambers and Glaucoma

**DOI:** 10.1155/2020/3690528

**Published:** 2020-11-16

**Authors:** Zhou Zhou, Li Li, Siming Zeng, Wenjing He, Min Li

**Affiliations:** ^1^Department of Ophthalmology, People's Hospital of Guangxi Zhuang Autonomous Region, Nanning 530021, Guangxi, China; ^2^Nanning Aier Eye Hospital, Nanning 530001, Guangxi, China

## Abstract

**Purpose:**

To compare femtosecond laser-assisted cataract surgery (FLACS) versus conventional phacoemulsification in shallow anterior chamber cataract patients with glaucoma or zonulysis.

**Methods:**

This was a single-center retrospective review of cataract surgeries in shallow anterior chamber and glaucoma patients between January 2016 and December 2018 in which a LenSx femtosecond laser was used. The outcome measures included pre- and postoperative uncorrected and corrected distance visual acuity (UDVA and CDVA), intraocular pressure (IOP), endothelial cell density (ECD), endothelial cell loss (ECL), and object scatter index (OSI).

**Results:**

One hundred and six eyes of 106 patients with a mean anterior chamber depth of 1.54 ± 0.51 mm were included in this study. Among them, 26 (23.2%) had zonulysis and 18 eyes had capsular tension ring implantation in general. The percentage of capsular tension ring implantation was statistically significantly lower in the FLACS group (*P* = 0.027). The UDVA, CDVA, ECD, and IOP were not statistically significant between the two groups at all time points. The postoperative ECL and OSI of the FLACS group was better than those of the conventional group (*P* < 0.01).

**Conclusions:**

FLACS can reduce ECL and improve visual quality compared to the conventional phacoemulsification in shallow anterior chamber patients. Also, it has the trend of reducing the use of capsular tension rings in subluxated cataracts. It is an ideal choice for patients with complicated cataract such as with shallow anterior chambers, glaucoma, and zonulysis.

## 1. Introduction

Femtosecond laser-assisted cataract surgery (FLACS) has become increasingly common since its introduction in 2009. Randomized controlled trials (RCTs) [1] and meta-analyses [2] have shown that it is not superior to manual phacoemulsification in terms of primary visual and refractive outcomes or overall complications. However, the benefits of FLACS, such as consistent and reproducible capsulotomy and nucleus fragmentation which result in less ultrasound energy required during phacoemulsification, may be especially advantageous in complex situations including shallow anterior chambers (ACs) and subluxated or white cataracts [3]. With growing experience in the use of femtosecond lasers, patients with heterogenous clinical features are being increasingly reported. Most of these are case reports or short case series. Recently, an RCT in India compared intraoperative performance and postoperative outcomes between FLACS and conventional phacoemulsification in eyes with a shallow AC and found that FLACS maintained clearer corneas, showed less increase in CCT, lower AC inflammation, and better UDVA in the early postoperative period [4]. However, the ACD average and range in that study were 2.33 mm and 2.1–2.5 mm. ACD is shallower in the Chinese population. The average ACD of Chinese healthy people was 2.89 ± 0.32 mm (range: 1.56–3.81 mm), [5] and that of angle-closure glaucoma patients was 1.86 ± 0.45 mm (range: 0.68–3.98 mm) [6]. Therefore, it is more meaningful to compare FLACS and conventional phacoemulsification in Chinese patients with shallow AC and angle-closure glaucoma. To explore the performance of femtosecond laser in patients with shallow anterior chamber, this study was conducted. The aim of the study is to evaluate the clinical outcomes of FLACS versus conventional phacoemulsification in eyes with shallow anterior chambers, glaucoma, or zonulysis and to compare their visual acuity and quality, IOP, and endothelial cell loss.

## 2. Methods

### 2.1. Design

This observational retrospective study was performed on patients who underwent cataract surgery at the Department of Ophthalmology of People's Hospital of Guangxi Zhuang Autonomous Region from January 2016 to December 2018. Informed consent was obtained from all subjects. This study was approved by the Institutional Review Board of People's Hospital of Guangxi Zhuang Autonomous Region and was conducted in accordance with contents of the Declaration of Helsinki.

### 2.2. Patients

After explaining the advantages and disadvantages of femtosecond laser-assisted surgery by the doctor, the patients chose the type of surgery by themselves. The patients were classified into two groups according to their operation methods. Inclusion criteria were symptomatic cataract for which the patient desires surgery and anterior chamber depth (ACD) less than 2.4 mm, with an elevated intraocular pressure of >21 mmHg. Exclusion criteria were <18 years of age, unable to give consent for surgery and research, and any other ocular diseases but glaucoma. Only one eye of each patient was included in the study, and in cases of bilateral cataracts, the eye undergoing surgery first was included.

### 2.3. Surgical Procedure

The surgical technique has been standardized for both groups. All surgeries were performed by the same experienced ophthalmologist. In the FLACS group, a 2.2 mm three-plane main incision, a 1.0 mm side-port corneal incision, a femtosecond laser-assisted capsulotomy (5.0 mm), and a six-piece lens division in a crisscross pattern were performed with a femtosecond laser (LenSx Lasers, Alcon Laboratories, Inc.). A 2.2 mm two-plane main incision and a 1.0 mm side-port corneal incision were made with a keratome in the conventional group, and capsule forceps were used to complete a 5.0 mm continuous curvilinear capsulorhexis in these patients. Both groups adopted phacoemulsification using the phaco-chop technique with the Centurion Vision Phacoemulsification System (Alcon Laboratories, Inc.). Intraoperatively, DisCoVisc (Alcon Laboratories, Inc.) was used to maintain the anterior chamber in all patients. All eyes in both groups accepted the single-piece, hydrophobic acrylic, aspheric intraocular lens (AcrySof SN60WF, Alcon Laboratories, Inc.).When finishing the phaco part and before implanting the IOL, if the dislocation of capsule is greater than one quadrant and less than two quadrants, we implanted a capsular tension ring.

### 2.4. Evaluation

Preoperatively, the medical histories of all patients were recorded. The uncorrected and corrected distance visual acuity (UDVA and CDVA) were evaluated by Snellen chart and documented in logarithm of the minimum angle of resolution (logMAR) units. Anterior chamber depth (ACD) measurement was performed using Scheimpflug imaging (Pentacam, Oculus Optikgerate GmbH) from the endothelial layer to the anterior lens surface. Postoperative visits occurred at day 1, week 2, and months 1, 3, and 6 and included a routine examination including UDVA, CDVA, and intraocular pressure (IOP) assessment and notation of any complications. Endothelial cell density (ECD) and object scatter index (OSI) were measured preoperatively and at 3 and 6 months postoperatively. A noncontact autofocus EM-3000 specular microscope (Tomey Corp.) was used to take endothelial photographs and to automatically count ECD values, and OSI was measured by using the Optical Quality Analysis System (OQAS, Visiometrics SL, Terrassa, Spain).

### 2.5. Statistical Analysis

Continuous data were conveyed by mean ± standard deviation and categorical data by number (percentage). Comparisons among the FLACS and conventional groups were analyzed using *t*-test for continuous variables and using a two-sided confidence interval of 95%. The chi-square test or Fisher's exact test was used for an association in four-fold table. Statistical analyses were performed with *R* project (version 3.4.2). The level of significance was set at a *P* value of less than 0.05 for all parameters.

## 3. Results

**Figure 1:**
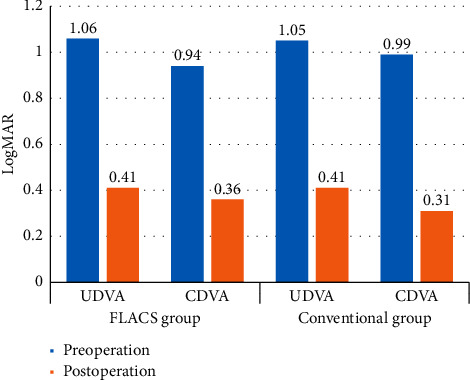
Pre- and postoperative UDVA and CDVA in the two groups. The postoperative LogMAR UDVA and CDVA were better than preoperation in both groups (*P* < 0.01).

A total of 106 eyes with 106 cataracts and shallow AC were included in this study, specifically 52 eyes in the FLACS group and 54 in the conventional group. Among all patients, 26 (24.5%) eyes had subluxated crystalline lenses, including 10 eyes in the FLACS group and 16 eyes in the conventional group. The range of lens dislocation was from 2 to 6 clock hours.  Table 1 shows the demographic data. Preoperatively, there was no statistically significant difference in age, gender composition, and ACD. The capsular tension rings (CTRs) were implanted in 18 (69.2%) eyes, including 4 eyes in the FLACS group and 14 eyes in the conventional group. The percentage of CTR implantation was statistically significantly lower in the FLACS group (*P* = 0.027). There were no intraoperative complications such as posterior capsule tear, vitreous loss, or Descemet's membrane detachment in either group.  Table 2 presents the pre- and postoperative UDVA, CDVA, and IOP values across the two groups at different time points. The postoperative UDVA and CDVA were better than preoperation in both groups (*P* < 0.01) (Figure 1). However, there was no statistically significant difference between the two groups. The groups' pre- and postoperative ECD and endothelial cell loss (ECL) values are shown in Table 3, and a statistically significant difference in ECL is evident (*P* < 0.001). As illustrated in Table 4, the OSI of the FLACS group at 3 and 6 months after surgery was statistically significantly better than that of the conventional group (*P* = 0.003).

## 4. Discussion

At present, femtosecond laser-assisted cataract surgery (FLACS) is being applied more and more in eyes with challenging cataracts or those with associated comorbidities, for example, dense or subluxated cataracts or Fuchs endothelial dystrophy, and this has revealed patients who are likely to benefit from the procedure. A shallow anterior chamber (AC) is a certain challenge for cataract surgeons worldwide, and extreme caution is needed when operating on such eyes because phacoemulsification will occur much closer to the endothelium, which increases the risk of endothelial cell loss.

One previous study has defined a shallow AC as an ACD less than 2.7 mm [7], and two others defined it as less than 2.5 mm [4, 8]. In order to include more patients, we defined a shallow AC as an ACD less than 2.5 mm. An RCT in India has previously compared FLACS with conventional phacoemulsification in eyes with shallow ACs [4]. The ACD average and range were 2.33 mm and 2.1–2.5 mm, respectively, in that study and 1.55 mm and 0.53–2.38 mm in ours. As such, ACD in the present study was shallower which makes our findings more representative of shallow AC patients.

Excessive ultrasound energy can lead to endothelial cell injury and cause early postoperative corneal edema which has itself been identified as the main cause of delayed visual rehabilitation and decreased satisfaction [9, 10]. Patients with shallow ACs are at higher risk of corneal endothelial cell injury [8, 11]. Previous studies have reported ECL from 4% to 12% after uneventful conventional phacoemulsification [12, 13]. The benefit of femtosecond laser in ordinary cataract patients is still unclear. An RCT found that the endothelial cell loss was 10.2% ± 13.7 in the FLACS group and 9.7 ± 13.7% in the conventional group (*P* = 0.76) [1]. However, two other RCTs, in Denmark [14] and Poland [15], showed that the ECL% was statistically significantly lower in the FLACS group postoperatively. FLACS had a statistically significant difference over conventional phacoemulsification for corneal endothelial cell reduction (*P* = 0.006) in a meta-analysis of 14, 567 eyes [2]. However, femtosecond laser may offer more help in high-risk cases. For example, FLACS has been found to be superior to phacoemulsification in reducing postoperative ECL in patients with Fuchs endothelial dystrophy, leading to a lower risk of corneal decompensation, particularly in patients with moderate or hard nucleus [16]. In our study, we observed no corneal endothelial decompensation and the mean ECL at 6 months was 8.40% and 12.86% in the FLACS group and conventional group, respectively. There was statistically significantly less ECL in the FLACS group (*P* < 0.001). Using a femtosecond laser can therefore be said to reduce intraoperative endothelial cell injury as compared to conventional phacoemulsification in angle-closure glaucoma and shallow AC patients.

In patients with glaucoma, it is common to see weak zonules, and in these cases, it is difficult to keep the capsule centered and to achieve precise sizing; the use of femtosecond lasers can solve these problems effectively. More precise capsulotomy sizing can be achieved with a femtosecond laser than in manual capsulorhexis, and femtosecond laser capsulotomies suffer less modification over time [17]. Using a femtosecond laser can ensure the centration and size of the capsulorhexis so that postoperative capsule contraction will not cause significant intraocular lens displacement. Fujikado and Saika found the increase in coma with decentration of aspheric IOLs suggesting an impact upon visual quality [18]. Miháltz et al. previously described a significant difference in IOL tilt between FLACS and conventional cohorts. Although there was no refractive difference, the authors note that coma was significantly less in the FLACS group, which implied the contribution of FLACS capsulorhexis in visual quality [19]. In our study, there was no statistical difference in postoperative visual acuity between the two groups. However, the OSI of the FLACS group at 3 and 6 months was statistically significantly better than that of the conventional group. Therefore, a FLACS approach can achieve better postoperative visual quality for angle-closure glaucoma and shallow AC patients.

For patients with zonulysis, femtosecond laser capsulorhexis can reduce the pulling of suspensory ligament during capsulorhexis, thus avoiding the expansion of dislocation range and reducing CTR implantation and even IOL suspension. Chee et al. treated patients with severely subluxated cataracts using femtosecond laser to perform the capsulotomy and nuclear fragmentation, preserving 90% capsular bags in eligible cases [20]. Ju et al. used femtosecond laser in the management of zonulysis, and 89.66% eyes showed stably centered IOLs [21]. Grewal et al. reported that FLACS helped a patient with traumatic subluxated cataract achieve a CDVA of 20/20 and a well-centered IOL [22]. Schultz et al. [23] and Crema et al. [24] reported that femtosecond laser-assisted cataract surgery helped Marfan syndrome patients gain a CDVA of 20/25∼20/20. In our study, the postoperative UDVA and CDVA were better than preoperation in both groups. The percentage of capsular tension ring implantation was statistically significantly lower in the FLACS group (*P* = 0.027). Our study showed the application of femtosecond laser technology increases the surgical safety and efficacy for zonulysis and helps to restore patients' visual function to the maximum extent.

The limitation of this study was that it was a retrospective study but not a randomized controlled trial. In order to fully respect the patient's right of choice, the type of surgery was chosen by the patients but not randomly assigned. However, after comparing the baseline of the two groups, we think the two groups are still comparable. Another limitation was that we did not measure the capsulorhexis or the position of IOL for it was a retrospective study. The next step should be comparing the quality of the capsulorhexis and the position of IOL between the two groups.

## 5. Conclusion

Femtosecond laser-assisted cataract surgery can help patients to get a better visual quality and reduce endothelial cell injury compared to the conventional phacoemulsification in shallow anterior chamber patients. It is an ideal choice for patients with complicated cataracts such as with shallow anterior chambers, angle-closure glaucoma, and zonulysis. It has significance for improving postoperative visual quality of these patients. Also, it has the trend of reducing the use of capsular tension rings in zonulysis cataracts.

## Figures and Tables

**Table 1:** Baseline demographics between the two groups.

	FLACS group	Conventional group	*P* value
Age (year)	67.19 ± 10.37	67.33 ± 8.84	0.958
Gender (female/male)	40 (76.92%)/12 (23.08%)	40 (74.07%)/14 (25.93%)	1
ACD (mm)	1.43 ± 0.62	1.64 ± 0.40	0.146
(i) Range	0.53 mm–2.38 mm	0.89 mm–2.36 mm	
With zonulysis	10 (19.2%)	16 (29.6%)	0.353
(ii) Preop subluxation range (o'clock)	3.20 ± 0.92	3.12 ± 0.96	0.845
(iii) CTR implantation	4 (40%)	14 (87.5%)	0.027^*∗*^

The values are represented as mean ± SD in age and ACD; number (percentage) in gender, with glaucoma, with subluxated lens, and CTR implantation. ACD, anterior chamber depth; CTR, capsular tension ring; FLACS, femtosecond laser-assisted cataract surgery. ^*∗*^Statistically significant.

**Table 2:** Pre- and postoperative UDVA, CDVA, and IOP between the two groups at different time points.

		Preop	Day 1	Week 2	Month 1	Month 3	Month 6
UDVA	FLACS group	1.06 ± 0.97	0.48 ± 0.54	0.49 ± 0.54	0.49 ± 0.54	0.46 ± 0.55	0.41 ± 0.48
	Conventional group	1.05 ± 0.93	0.47 ± 0.40	0.44 ± 0.33	0.44 ± 0.34	0.41 ± 0.32	0.41 ± 0.32
	*P* value	0.989	0.959	0.72	0.668	0.64	0.988

CDVA	FLACS group	0.94 ± 1.00	0.44 ± 0.57	0.44 ± 0.57	0.42 ± 0.58	0.40 ± 0.59	0.36 ± 0.51
	Conventional group	0.99 ± 0.95	0.40 ± 0.43	0.36 ± 0.35	0.35 ± 0.37	0.31 ± 0.35	0.31 ± 0.35
	*P* value	0.847	0.795	0.539	0.564	0.468	0.666

IOP	FLACS group	20.76 ± 4.15	12.87 ± 2.63	13.91 ± 3.10	13.98 ± 2.38	14.45 ± 2.21	14.84 ± 2.87
	Conventional group	21.16 ± 5.76	13.55 ± 2.90	13.79 ± 3.13	14.25 ± 2.88	13.88 ± 2.92	14.51 ± 2.27
	*P* value	0.767	0.364	0.882	0.714	0.422	0.627

The values are represented as mean ± SD. UDVA, uncorrected distance visual acuity; CDVA, corrected distance visual acuity; IOP, intraocular pressure; FLACS, femtosecond laser-assisted cataract surgery.

**Table 3:** Pre- and postoperative operative ECD between the two groups at different time points.

	Preoperation	Month 3	Month 6	ECL at month 6	ECL%
FLACS group	2185.51 ± 666.55	2022.07 ± 622.69	2001.85 ± 616.47	183.66 ± 70.83	8.40%
Conventional group	2473.25 ± 679.83	2176.85 ± 570.98	2155.09 ± 565.27	318.16 ± 130.07	12.86%
*P* value	0.117	0.336	0.336	<0.001^*∗*^	<0.001^*∗*^

The values are represented as mean ± SD. ECD, endothelial cell density; ECL, endothelial cell loss; FLACS, femtosecond laser-assisted cataract surgery. ^*∗*^Statistically significant.

**Table 4:** Pre- and postoperative OSI between the two groups at different time points.

	Preop	Month 3	Month 6
FLACS group	3.51 ± 1.64	1.95 ± 0.57	1.88 ± 0.54
Conventional group	4.30 ± 1.97	2.41 ± 0.53	2.34 ± 0.54
*P* value	0.111	0.003^*∗*^	0.003^*∗*^

The values are represented as mean ± SD. OSI, object scatter index; FLACS, femtosecond laser-assisted cataract surgery. ^*∗*^Statistically significant.

## Data Availability

The data used to support the findings of this study are included within the article.

## Abbreviations

AC: Anterior chamber
ACD: Anterior chamber depth
CDVA: Corrected distance visual acuity
CTR: Capsular tension ring
ECD: Endothelial cell density
FLACS: Femtosecond laser-assisted cataract surgery
IOL: Intraocular lens
IOP: Intraocular pressure
logMAR: Logarithm of the minimum angle of resolution
OSI: Object scatter index.
Phaco: Phacoemulsification
SD: Standard deviation
UDVA: Uncorrected distance visual acuity.
